# Stains for Fat

**Published:** 1907-12-07

**Authors:** 


### Clinical Methods.

### STAINS FOR FAT.

It often happens that one wishes to know whether a particular specimen one is examining under the microscope contains fat droplets or not. It is frequently of importance to know whether there is any excess of fat in the fæces, as an indication of possible deficiency in the secretion of either bile or pancreatic juice, or both; in the examination of milk it may be of great assistance to one if the fat globules can be stained in some distinctive way so that they can at once be distinguished from any cellular elements that may be present: the same applies to gastric contents that are being examined for cells or particles of new growth. If the fat globules can be stained one colour, and the tissue cells another, the examination is greatly simplified. Sometimes, again, it is important to know whether the fine particles that may be present in an ascitic fluid consist of fat or not, the presence of any considerable quantity of fat being evidence of obstruction to the lymphatics, in addition to any backward pressure from the heart or other cause for the ascites that exists. In this country chyluria is not common, though it is not unknown; but abroad it is not uncommon as the result of filariasis, and it may be of considerable diagnostic importance to know whether any of the microscopical granules that may be seen in the urine are fat or not. In some cases of diabetes a condition of lipæmia sets in previous to coma—a warning that coma is imminent—and the fat particles in the blood are in such a fine state of subdivision that they are not easily recognisable unless they can be picked out by some distinctive stain.

It is clear, therefore, that there are many different kinds of cases in which a good stain for fat is likely to be of use in assisting diagnosis. The best-known stain for the purpose is osmic acid. It is mainly because this is the one most often employed that this note upon the subject is written; for osmic acid, though best known, is by no means actually the best stain for the purpose. It has the disadvantage of not keeping well, for one thing; it must be protected from exposure to daylight if it is to be kept at all, and even then it decomposes and deposits a black precipitate. In the second place, osmic acid does not always stain fat droplets with great rapidity, so that there is a danger of concluding that there is no fat present when the fluid or the tissue is examined with anything like haste. In the third place, osmic acid will stain some droplets and particles which are not really fat a dark brown or dark green colour, so that one may conclude that they are fat when they are really something else; finally, when the particles of fat are very fine indeed, as in chyluria, chylous ascites, the early stages of acute fatty degeneration of the heart muscle, and so forth, it is often very difficult indeed to be sure whether they have been stained black or not.

There are two other dyes that have been strongly recommended by Dudgeon, Shattock, and others in preference to osmic acid—namely, (1) Scharlach R., and (2) Sudan III. These are both obtainable in the solid form at a small cost, and they can be readily made up into the required solutions; or, if preferred, the solutions can be bought ready made up from any of the firms in London or elsewhere who deal with microscopical stains. The dyes themselves are of Grübler's manufacture, and they are imported in powder form in glass tubes containing from 5 grammes upwards. The cost of each tube of 5 grammes is a few pence.

The names Scharlach R. and Sudan III. are those by which the stains are known and sold; their chemical composition appears to be as follows:—

Scharlach R = azo-orthotoluol-azo-$\beta$-naphthol.

Sudan III. = azo-benzine-azo-$\beta$-naphthol.

so that they are related to one another. The solution of each is a bright red colour, and when mixed with the fluid to be examined, or poured on to the section to be stained, the fat droplets or particles almost immediately turn brilliant red, whilst other structures either do not take up the stain at all, or at any rate do not do so with anything like the avidity that fat does. The dyes keep well, and they are very easy to use; hence for the detection of fat in pathological fluids or tissues either Scharlach R. or Sudan III. is better in almost all respects than is osmic acid.

